# Are exhaled nitric oxide measurements using the portable NIOX MINO repeatable?

**DOI:** 10.1186/1465-9921-11-43

**Published:** 2010-04-23

**Authors:** Anna Selby, Bernie Clayton, Jane Grundy, Katy Pike, Kirsty Drew, Abid Raza, Ramesh Kurukulaaratchy, S Hasan Arshad, Graham Roberts

**Affiliations:** 1 School of Medicine, University of Southampton, Southampton, UK; 2 The David Hide Asthma and Allergy Research Centre, St Mary's Hospital, Newport, Isle of Wight, UK; 3 Southampton University Hospital NHS Trust, Southampton, UK

## Abstract

**Background:**

Exhaled nitric oxide is a non-invasive marker of airway inflammation and a portable analyser, the NIOX MINO (Aerocrine AB, Solna, Sweden), is now available. This study aimed to assess the reproducibility of the NIOX MINO measurements across age, sex and lung function for both absolute and categorical exhaled nitric oxide values in two distinct groups of children and teenagers.

**Methods:**

Paired exhaled nitric oxide readings were obtained from 494 teenagers, aged 16-18 years, enrolled in an unselected birth cohort and 65 young people, aged 6-17 years, with asthma enrolled in an interventional asthma management study.

**Results:**

The birth cohort participants showed a high degree of variability between first and second exhaled nitric oxide readings (mean intra-participant difference 1.37 ppb, 95% limits of agreement -7.61 to 10.34 ppb), although there was very close agreement when values were categorised as low, normal, intermediate or high (kappa = 0.907, p < 0.001). Similar findings were seen in subgroup analyses by sex, lung function and asthma status. Similar findings were seen in the interventional study participants.

**Conclusions:**

The reproducibility of exhaled nitric oxide is poor for absolute values but acceptable when values are categorised as low, normal, intermediate or high in children and teenagers. One measurement is therefore sufficient when using categorical exhaled nitric oxide values to direct asthma management but a mean of at least two measurements is required for absolute values.

## Introduction

Asthma is a chronic inflammatory disorder of the airways associated with airway hyperresponsiveness and recurrent episodes of reversible airflow limitation that are accompanied by wheeze, shortness of breath, chest tightness and cough [1]. It is the most common chronic condition of childhood [2] affecting approximately 20% of school-aged children in the United Kingdom [3]. Decisions regarding asthma management are currently based on symptoms and conventional lung function tests. Exhaled nitric oxide (FeNO) has recently emerged as a potentially useful tool in the assessment of patients with asthma [4]. Exhaled nitric oxide measurements correlate well with measures of airway inflammation, including sputum levels of eosinophils [5], airway eosinophilia in bronchial biopsies [6] and allergen exposure [7]. Measurements can be made within minutes, even in young school children. Furthermore, FeNO has been shown to rise prior to asthma exacerbations [8] and decrease following administration of inhaled corticosteroids [9] or leukotriene receptor antagonists [10]. Potential applications for FeNO may therefore be found in the diagnostic work up of patients with possible asthma, monitoring of treatment responses, adherence with treatment and prediction of exacerbations [4,11]. Its role in directing the chronic management of asthma has though been questioned recently [12,13]. Previously, FeNO measurement required static, chemiluminesence-based NO analysers, such as the NIOX Nitric Oxide Monitoring System (Aerocrine AB, Solna, Sweden). The use of these analysers in routine clinical practice is, however, limited by their size and expense. A portable hand-held NO-analyser, the NIOX MINO Asthma Inflammation Monitor (Aerocrine AB, Solna, Sweden), which uses electrochemical sensors to measure FeNO levels, is now available. This is ideally suited for use in primary care, where the majority of asthma patients are managed [14].

Studies comparing the performance of the NIOX MINO and the NIOX have shown that the level of agreement between the two devices is clinically acceptable [15-18] and the NIOX MINO is now approved by the Federal Drugs Administration in the United States for assessing asthma-related airway inflammation [19]. The manufacturer recommends that one measurement is sufficient when using the NIOX MINO [20] rather than two as recommended in the latest ATS/ERS guidelines [4]. The manufacturer states an accuracy of ±5 ppb of measured value below 50 ppb and ±10% at or above 50 ppb [20]. There are a number of studies that support this recommendation of a single measurement [15,16,21,22], however, to date only one small paediatric study has addressed this question [18]. In this study of fifty-five children aged 4-15 years, the first acceptable FeNO measurement obtained using the NIOX MINO was not significantly different from the mean of all measurements (24 versus 27 ppb, p > 0.5) but the median coefficient of variation for the NIOX MINO was 7.4% (range: 0-44.6) suggesting that one FeNO measurement is not sufficient when using the NIOX MINO. The aim of the current validation study was to assess the reproducibility of NIOX MINO measurements in children and teenagers across age, sex and lung function, in terms of both the absolute and categorical FeNO values.

## Methods

### Study design and participants

In this validation study, the reproducibility of NIOX MINO measurements was assessed in two separate populations.

#### Unselected, community-based, birth cohort

The birth cohort consisting of teenagers aged 16-18 years enrolled in a whole population birth cohort (Research ethics reference 06/Q1701/34) of 1536 infants born on the Isle of Wight, United Kingdom in 1989/1990 to investigate the natural history of asthma and allergic disorders. All these teenagers were assessed with questionnaires, FeNO measurements, spirometry and skin prick testing. In accordance with the ATS/ERS guidelines [4], FeNO measurements (NIOX MINO, Aerocrine AB, Solna, Sweden) were performed prior to spirometric testing with participants standing. The NIOX MINO 300 sensor was used with the sensor being changed after 300 measurements. Participants were asked to inhale to total lung capacity through the NIOX MINO and then exhale for 10 seconds at 50 ml/sec (assisted by visual and auditory cues). Spirometry (KoKo, nSpire Health, Hertford, United Kingdom) was performed according to the ATS/ERS guidelines [23]. FEV_1 _was recorded as percent predicted for age, height, sex and ethnic origin. Skin prick testing was performed by a standardised method [24] to a panel of common allergens: house dust mite (Dermatophagoides pteronyssinus), grass pollen mix, tree pollen mix, cat and dog epithelia, Alternaria alternata, Cladosporium herbarum, milk, hens' egg, wheat, soya, cod and peanut as well as histamine and physiological saline (Alk-Abello, Horsholm, Denmark). Single-headed lancets were used and the skin pricked at an angle of 90°. The wheal diameter was recorded at 15 minutes.

#### Interventional asthma study

This multi-centre study was designed to investigate whether monitoring FeNO levels can improve the management of children with asthma (Research ethics reference 06/Q1702/9). The study sites were Southampton University Hospital NHS Trust, St Mary's Hospital in Newport on the Isle of Wight and St Mary's Hospital in Portsmouth. Inclusion criteria for the interventional study were age 6-17 years, clinical diagnosis of asthma (based on typical symptoms, at least a 15% increase in FEV_1 _(forced expiratory volume in 1 second) with bronchodilator or at least 15% diurnal variability in PEF rates) and receiving treatment with at least 400 mcg beclomethasone equivalent daily. Exclusion criteria were cigarette smoking, poor adherence with medication, previous life-threatening exacerbations or the need for maintenance oral prednisolone. In the intervention study, participants were assessed 2-monthly for a year. The spirometry data (KoKo, nSpire Health, Hertford, United Kingdom) and two FeNO measurements (NIOX MINO, Aerocrine AB, Solna, Sweden) used in this study were obtained at the same visit. Both were measured as per the cohort participants. Demographic details, asthma history, asthma treatment and history of other atopic diseases were recorded for all participants. Skin prick testing was performed as per the cohort participants to a grass pollen mix, tree pollen mix, cat, dog, house dust mite, saline and histamine (ALK-Abello, Horsholm, Denmark). A single wheal of at least 3 mm was considered indicative of atopy in the presence of appropriate negative and positive control results.

### Statistical Analysis

Data were transferred to SPSS version 15 for analysis. Bland-Altman plots were constructed to assess the degree of agreement between the absolute values of paired FeNO readings measured using the NIOX MINO. FeNO were logarithmically transformed to normalise the data. Cohen's Kappa was used to assess the degree of agreement between paired categorical NIOX MINO measurements. Nitric oxide values were categorised as low, normal, intermediate or high according to the reference ranges for age less than 12 years and 12 years or more provided by Aerocrine [25] (Table 1). Subgroup analyses were undertaken for males and females, participants with low and high FEV_1 _values, older and younger participants and participants with and without asthma.

**Table 1:** Guide to interpreting FeNO values.

	FeNO (ppb)	
	
	Children (<12 years)	Adults (≥12 years)
**Low**	<5	<5

**Normal**	5-20	5-25

**Intermediate**	20-35	25-50

**High**	>35	>50

Categories based on Taylor et al [25,27].

## Results

### Birth cohort

#### Study participants

Paired NIOX MINO measurements were obtained from 494 of the participants enrolled in the 17 year follow-up study up to 11^th ^December 2007. Of these, 71 were classified as having current asthma on the basis of positive responses to both ISAAC validated questions 'Have you ever been diagnosed with asthma by a physician?', and, 'Have you had wheezing or whistling in the chest in the last 12 months?'. The demographic and clinical characteristics of the participants in this population alongside those of the intervention study participants are shown in Table 2.

**Table 2:** Clinical and demographic characteristics of the study participants.

	Birth cohort participants*			
		
	All participants	Participants with asthma	Non-asthmatic participants	Intervention study participants
	(n = 494)	(n = 71)	(n = 401)	(n = 65)
**Age (years), mean (range)**	17.17 (16-18)	17.21 (16-18)	17.16 (16-18)	11 (6-16)

**Sex, no. (%)**				

**Male**	251 (50.8)	36 (50.7)	207 (51.6)	38 (58.5)

**Female**	243 (49.2)	35 (49.3)	194 (48.4)	27 (41.5)

**Hay fever, no. (%)**	183 (37.0)	49 (69.0)	125 (31.2)	56 (86.2)

**Eczema, no. (%)**	82 (16.6)	24 (33.8)	56 (14.0)	38 (58.5)

Atopy (as defined by skin prick testing), no. (%) **	195 (42.1)	48 (72.7)	141 (37.3)	37/42 (88.1)

**FEV**_**1**_**(% predicted), mean (SD)**	104.58 (13.22)	97.34 (15.38)	105.99 (12.28)	90.35 (14.57)

FeNO (ppb), median (interquartile range) ***	16 (11-31)	40 (16-74)	15 (11-25.5)	35 (13.5-57.0)

**Asthma treatment, no. (%)**				
**Inhaled corticosteroids**	NA	34 (47.9)	NA	65 (100)
				
**Short-acting bronchodilators**		46 (64.8)		65 (100)
				
**Long-acting bronchodilators**		12 (16.9)		48 (73.8)
				
**Montelukast**		1 (1.4)		36 (55.4)
				
**Theophylline**		0 (0)		3 (4.6)

*Some of the birth cohort participants could not be categorised because not all questions were answered. NA: Not applicable.

** Percentages are based on the number of participants in whom skin prick testing was performed.

*** Values are based on the first FeNO reading obtained in each participant.

#### Reproducibility of the absolute value of FeNO

**Figure 1:**
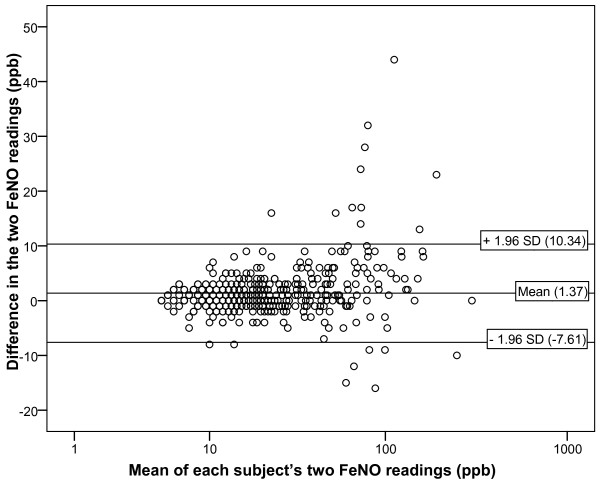
**Bland Altman plot showing the difference between each subject's two FeNO NIOX MINO measurements in the birth cohort**. Each point represents the absolute difference between the first and second FeNO measurements for each participant versus the mean of these two measurements (n = 494). Reference lines correspond to the mean difference in two FeNO measurements taken in one individual and the 95%.

Although a statistically significant linear association was found between the first and second FeNO readings in individual participants (Pearson's correlation coefficient = 0.980, p < 0.001), a Bland-Altman plot showed a high degree of variability between these paired NIOX MINO measurements (Figure 1). The mean intra-participant difference in FeNO (second FeNO reading minus first FeNO reading) was 1.37 ppb, suggesting that the second FeNO reading in each participant was on average higher than the first. This difference was statistically different from zero (one sample t-test, p < 0.001). The 95% limits of agreement of -7.61 to 10.34 ppb imply that if two NIOX MINO measurements are undertaken in the same assessment, there is a 95% chance that the second FeNO value will be up to 10 ppb above or 8 ppb below the first. Much of this variability occurs at higher FeNO levels (Figure 1) and, when subjects with FeNO values above 75 ppb were excluded from the analysis, the mean intra-participant difference in FeNO was 0.90 ppb and the 95% limits of agreement were -4.89 to 6.70 ppb. Similar findings were seen in subgroup analyses for males and females, participants with an FEV_1 _in the lowest and highest tertiles, and participants with and without asthma (Table 3). In participants with asthma, the mean intra-participant difference in FeNO was 2.37 ppb and the 95% limits of agreement were -11.38 to 16.12 ppb.

**Table 3:** Subgroup analyses of the reproducibility of the absolute value of FeNO in the birth cohort participants.

	Mean intra-participant difference in FeNO (ppb) (95% CI) [p-value] *	Standard deviation (SD) of the intra-participant difference in FeNO (ppb)	95% limits of agreement (ppb)
**All participants**	1.37	4.58	-7.61 to 10.34
**(n = 494)**	(0.96, 1.77) [<0.001]		

**Males**	1.43	4.97	-8.30 to 11.17
**(n = 251)**	(0.82, 2.05) [<0.001]		

**Females**	1.30	4.15	-6.83 to 9.43
**(n = 243)**	(0.77, 1.82) [<0.001]		

**Participants with a low FEV**_**1**_	2.01	5.21	-8.20 to 12.22
**(n = 162)**	(1.20, 2.81) [<0.001]		

**Participants with a high FEV**_**1**_	1.07	3.75	-6.28 to 8.42
**(n = 162)**	(0.49, 1.65) [<0.001]		

**Participants with asthma**	2.37	7.01	-11.38 to 16.12
**(n = 71)**	(0.71, 4.03) [0.006]		

**All others participants**	1.19	4.09	-6.82 to 9.20
**(n = 401)**	(0.79, 1.59) [<0.001]		

A low FEV_1 _implies an FEV_1_in the lowest tercile for the birth cohort participants (less than 98.94% predicted), whilst a high FEV_1 _implies an FEV_1_in the highest tercile for all the birth cohort participants (greater than 109.80% predicted).

95% limits of agreement = Mean intra-participant difference in FeNO ± 1.96 (SD)

* For comparison of the mean intra-participant difference in FeNO in each group against zero (one sample t-test).

#### Reproducibility of categorical FeNO values

When FeNO values were categorised as low, normal, intermediate or high (using <12 or ≥12 year values as appropriate), very close agreement between paired NIOX MINO measurements in individual participants was observed (Kappa (κ) = 0.907, p < 0.001) (Table 4). In the planned subgroup analyses, very close agreement was seen in males (κ = 0.894, p < 0.001) and females (κ = 0.919, p < 0.001), participants with an FEV_1 _in the lowest (κ = 0.943, p < 0.001) and highest (κ = 0.872, p < 0.001) tertiles, and in those with current asthma (κ = 0.935, p < 0.001).

**Table 4:** Agreement between paired NIOX MINO measurements in the birth cohort participants when FeNO values were categorised as low, normal, intermediate or high.

	First FeNO reading				
		
	Low	Normal	Intermediate	High	Total
**Second FeNO reading**					

**Low**	0 (0%)	0 (0%)	0 (0%)	0 (0%)	**0 (0%)**

**Normal**	0 (0%)	334 (97%)	3 (4%)	0 (0%)	**337 (68%)**

**Intermediate**	0 (0%)	10 (3%))	72 (87%)	1 (2%)	**83 (17%)**

**High**	0 (0%)	0 (0%)	8 (10%)	66 (99%)	**74 (15%)**

**Total**	**0 (0%)**	**344 (100%)**	**83 (100%)**	**67 (100%)**	**494 (100%)**

Cohen's Kappa (κ) = 0.907 (p < 0.001)

Figures represent numbers of participants (% by column). Categories are presented in Table 1.

### Intervention study

Paired FeNO readings were obtained from 65 of the participants enrolled in the intervention study. The characteristics of these participants are outlined in Table 2, all had typical symptoms of asthma. As expected, these participants had more severe asthma and were more likely to be atopic.

#### Reproducibility of the absolute value of FeNO

**Figure 2:**
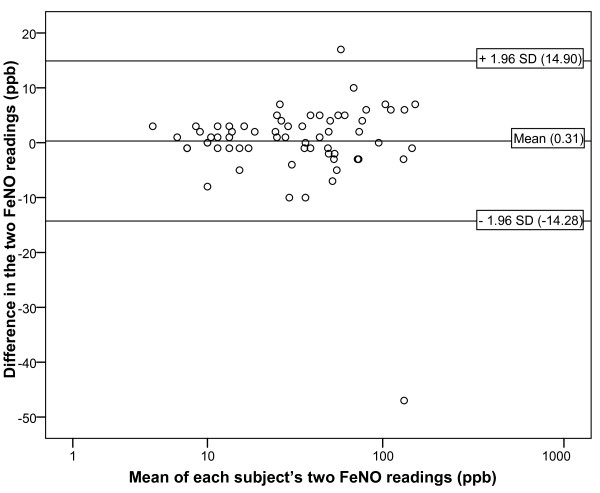
**Bland Altman plot showing the difference between each subject's two FeNO NIOX MINO measurements in the intervention study**. Each point represents the absolute difference between the first and second FeNO measurements for each participant versus the mean of these two measurements (n = 65). Reference lines correspond to the mean difference in two FeNO measurements taken in one individual and the 95% limits of agreement. The normal range of FeNO measurements is 5 to 20 ppb in children <12 years and 5 to 25 ppb in teenagers and adults.

Once again, there was a statistically significant linear association between the first and second FeNO readings in individual participants (Pearson's correlation coefficient = 0.977, p < 0.001). Although the mean intra-participant difference in FeNO for all participants was only 0.31 ppb, which was not different from zero (one sample t-test, p = 0.740), the 95% limits of agreement were widely spread (-14.28 to 14.90 ppb) (Figure 2). Even wider limits of agreement were observed in males, participants in the highest tertile for age and participants with an FEV_1 _in the lowest tertile (Table 5). When FeNO values above 75 ppb were excluded the intra-participant difference in FeNO was 0.64 ppb with 95% limits of agreement of -8.35 to 9.62 ppb.

**Table 5:** Subgroup analyses of the reproducibility of the absolute value of FeNO in the intervention study participants.

	Mean intraparticipant difference in FeNO (ppb) (95% CI) [p-value] *	Standard deviation (SD) of the intra-participant difference in FeNO (ppb)	95% limits of agreement (ppb)
**All participants**	0.31	7.45	-14.28 to 14.90
**(n = 65)**	(1.54, 2.15) [0.740]		

**Males**	-0.26	9.39	-18.66 to 18.14
**(n = 38)**	(-3.35, 2.82) [0.864]		

**Females**	1.11	3.14	-5.05 to 7.27
**(n = 27)**	(-0.13, 2.35) [0.078]		

**Youngest participants**	-0.09	4.08	-8.09 to 7.91
**(n = 22)**	(-1.9, 1.72) [0.918]		

**Oldest participants**	1.10	12.07	-22.55 to 24.75
**(n = 21)**	(-4.40, 6.89) [0.682]		

**Participants with a low FEV**_1_	-1.45	11.19	-23.38 to 20.48
**(n = 22)**	(-6.42, 3.51) [0.549]		

**Participants with a high FEV**_**1**_	0.15	3.28	-6.28 to 6.58
**(n = 20)**	(-1.39, 1.69) [0.840]		

A low FEV_1 _implies an FEV_1_within the lowest tercile for the intervention study participants (less than 83% predicted), whilst a high FEV_1 _implies an FEV_1_within the highest tercile for the intervention study participants (greater than 95% predicted).

The youngest participants are those within the lowest tercile for age (less than 10 years 7 months), whilst the oldest participants are those within the highest tercile for age (greater than 12 years 8 months).

95% limits of agreement = Mean intra-participant difference in FeNO ± 1.96 (SD)

* For comparison of the mean intra-participant difference in FeNO in each group against zero (one sample t-test).

#### Reproducibility of categorical FeNO values

For all participants, a Kappa value (κ) of 0.879 (p < 0.001) was ascertained, suggesting very close agreement between the first and second FeNO readings in individual participants when FeNO values were categorised as low, normal, intermediate or high (Table 6). A similarly high degree of agreement between categorical FeNO values was observed in males (κ = 0.823, p < 0.001) and females (κ = 0.940, p < 0.001), participants in the lowest tertile for age (κ = 0.921, p < 0.001) and participants with an FEV_1 _in the lowest (κ = 0.845, p < 0.001) and highest (κ = 1.000, p < 0.001) tertiles. It was lower for participants in the highest tertile for age (κ = 0.667, p < 0.001), although even in this group, agreement was reasonable.

**Table 6:** Agreement between paired NIOX MINO measurements in the intervention study participants when FeNO values were categorised as low, normal, intermediate or high.

	First FeNO reading				
		
	Low	Normal	Intermediate	High	Total
**Second FeNO reading**					

**Low**	0 (0%)	0 (0%)	0 (0%)	0 (0%)	**0 (0%)**

**Normal**	1 (100%)	22 (96%)	0 (0%)	0 (0%)	**23 (35%)**

**Intermediate**	0 (0%)	1 (4%)	12 (80%)	1 (4%)	**14 (22%)**

**High**	0 (0%)	0 (0%)	3 (20%)	25 (96%)	**28 (43%)**

**Total**	**1 (100%)**	**23 (100%)**	**15 (100%)**	**26 (100%)**	**65 (100%)**

Cohen's Kappa (κ) = 0.879 (p < 0.001)

Figures represent numbers of participants (% by column). Categories are presented in Table 1 with the <12 or ≥12 year categorical values being used as appropriate for individual participants.

## Discussion

This study aimed to validate the reproducibility of a new, portable NO-analyser (the NIOX MINO, Aerocrine AB, Solna, Sweden) in children and teenagers. The results from two complementary groups of children and teenagers indicate that the reproducibility of NIOX MINO measurements across the paediatric age range is poor when considering the absolute value FeNO but is acceptable when FeNO values are categorised as low, normal, intermediate or high. This suggests that if clinical decisions in future are based on specific cut-off values of FeNO, the mean of at least two FeNO measurements should be reported when using the NIOX MINO. However, for treatment algorithms based on FeNO categories, one NIOX MINO measurement would be sufficient, saving cost and time in clinic. This study is the largest of its kind to date and is the first to consider the effect of patient characteristics on reproducibility of NIOX MINO measurements.

Our findings contradict those of other studies, which have reported excellent reproducibility of the absolute value of FeNO measurements obtained using the NIOX MINO [15,16,21,22]. These studies have, however, included both adults and children, so their results do not validate the use of the NIOX MINO in paediatric practice. Much of this intra-subject variability is seen at higher levels of FeNO and so it may be more patient related than monitor related [26] and unlikely to cause any indecision about clinical management. For the intervention asthma study participants, the reproducibility of the absolute value of FeNO was poorer in those with a low FEV_1 _whilst in the birth cohort participants, greater variability was observed in participants with asthma. These are precisely the participants in whom FeNO measurement would be performed in clinical practice. Our results therefore support McGill's recommendation that the mean of at least two FeNO measurements should be used when reporting absolute values using the NIOX MINO with children and teenagers [18]. It has been suggested that greater variability between FeNO measurements is more likely in those who are not consistently able to record FeNO [18]. Given that the success rate with the NIOX MINO increases with age [15], it is therefore surprising that we observed closer agreement between paired NIOX MINO measurements in the youngest participants (Table 5). Potentially, taking the average of three or more measurements may have further increased the reliability of the FeNO value but this was outside the scope of this study. The other potential limitation of this study is that a gold standard measure of FeNO, such as the NIOX monitor (Aerocrine AB, Solna, Sweden) was not included; again this was outside the scope of this study which was focusing on the reproducibility of NIOX MINO readings within one assessment rather than their validity.

No previous studies have evaluated the reproducibility of categorical FeNO values obtained using the NIOX MINO. Our data suggests that the reproducibility of this approach is acceptable although 10% of subjects may be misclassified by one category. These categories are based on cut off values from a number of studies [25,27] and there may be a certain amount of imprecision in the boundaries chosen. The small amount of misclassification bias is likely to be of minimal clinical importance unless FeNO levels are being used in isolation to diagnose asthma [28] or direct the clinical management of patients with asthma [4]. Different research groups are, however, using different cut off values, so it is important to assess whether reproducibility is comparable with other cut off values. Additionally, future research should seek to determine whether the accuracy of NIOX MINO measurements is affected by the number of attempts required to record FeNO, as this would have implications for the interpretation of FeNO measurements in clinical practice. This is particularly important for patients less than 12 years of age who often need more attempts and were relatively poorly represented in our study. Lastly, given the number of influences on FeNO levels, these measurement may proved to be most useful when standardised to the patient's baseline FeNO level while their asthma is well controlled as we have previously suggested [7]. Such a strategy would necessitate a further look at the consequences of relying on a single estimate of the level of FeNO in driving clinical management.

In summary, the NIOX MINO could improve the management of children with asthma by enabling physicians to monitor underlying airway inflammation more easily than has been possible to date. According to the manufacturer (Aerocrine AB, Solna, Sweden), one FeNO measurement is sufficient when using the NIOX MINO, instead of two as recommended by the ATS/ERS guidelines. This study has found that in two distinct groups of children and teenagers, one NIOX MINO measurement is acceptable when using FeNO to direct asthma management, based on FeNO categories, but when using the absolute value of FeNO, the mean of at least two NIOX MINO measurements should be used.

## Competing interests

The authors declare that they have no competing interests.

## Authors' contributions

AS: design, acquisition of data, analysis and interpretation, manuscript writing; BC: acquisition of data and critical revision of manuscript; JG: acquisition of data and critical revision of manuscript; KP: acquisition of data, analysis and critical revision of manuscript; KD: acquisition of data and critical revision of manuscript; AR: acquisition of data and critical revision of manuscript; RK: interpretation of data and critical revision of manuscript; SHA: interpretation of data and critical revision of manuscript; GR: conception anddesign, analysis and interpretation, critical revision and final approval. All authors have read and approved the final manuscript.

## References

[B1] GINA Global Strategy for Asthma Management and Prevention 2004

[B2] von Mutius E The burden of childhood asthma Arch Dis Child 2000 82 Suppl II ii2 ii5 10833469 10.1136/adc.82.suppl_2.ii2 PMC1765082

[B3] The International Study of Asthma and Allergies in Childhood (ISAAC) steering committee Worldwide variations in the prevalence of asthma symptoms: the International Study of Asthma and Allergies in Childhood (ISAAC) Eur Resp J 1998 12 315 35 10.1183/09031936.98.12020315 9727780

[B4] American Thoracic Society, European Respiratory Society ATS/ERS Recommendations for standardized procedures for the online and offline measurement of exhaled lower respiratory nitric oxide and nasal nitric oxide Am J Resp Crit Care Med 2005 171 912 30 10.1164/rccm.200406-710ST 15817806

[B5] Jatakanon A Lim S Kharitonov SA Chung KF Barnes PJ Correlation between exhaled nitric oxide, sputum eosinophils, and methacholine responsiveness in patients with mild asthma Thorax 1998 53 91 5 10.1136/thx.53.2.91 9624291 PMC1758706

[B6] Payne D Adcock I Wilson N Oates T Scallan M Bush A Relationship between exhaled nitric oxide and mucosal eosinophilic inflammation in children with difficult asthma, after treatment with oral prednisolone Am J Respir Crit Care Med 2001 164 1376 81 11704581 10.1164/ajrccm.164.8.2101145

[B7] Roberts G Hurley C Bush A Lack G Longitudinal study of grass pollen exposure, seasonal allergic asthma symptoms, and exhaled nitric oxide in childhood seasonal allergic asthma Thorax 2004 59 752 6 10.1136/thx.2003.008722 15333850 PMC1747120

[B8] Jones SL Kittelson J Cowan JO Flannery EM Hancox RJ McLachlan CR Taylor DR The Predictive value of exhaled nitric oxide measurements in assessing changes in asthma control Am J Respir Crit Care Med 2001 164 738 43 11549525 10.1164/ajrccm.164.5.2012125

[B9] Jones SL Herbison P Cowan JO Flannery EM Hancox RJ McLachlan CR Taylor DR Exhaled NO and assessment of anti-inflammatory effects of inhaled steroid: dose-response relationship Eur Respir J 2002 20 601 8 10.1183/09031936.02.00285302 12358335

[B10] Montuschi P Mondino C Koch P Ciabattoni G Barnes PJ Baviera G Effects of montelukast treatment and withdrawal on fractional exhaled nitric oxide and lung function in children with asthma Chest 2007 132 1876 81 10.1378/chest.07-1587 18079221

[B11] Zacharasiewicz A Wilson N Lex C Erin EM Li AM Hansel T Khan M Bush A Clinical use of noninvasive measurements of airway inflammation in steroid reduction in children Am J Respir Crit Care Med 2005 171 1077 1082 10.1164/rccm.200409-1242OC 15709050

[B12] Szefler SJ Mitchell H Sorkness CA Gergen PJ O'Connor GT Morgan WJ Kattan M Pongracic JA Teach SJ Bloomberg GR Eggleston PA Gruchalla RS Kercsmar CM Liu AH Wildfire JJ Curry MD Busse WW Management of asthma based on exhaled nitric oxide in addition to guideline-based treatment for inner-city adolescents and young adults: a randomised controlled trial Am J Resp Crit Care Medicine 2008 372 1065 1072 10.1016/S0140-6736(08)61448-8 PMC2610850 18805335

[B13] Jongste JC Carraro S Hop WC CHARISM Study Group Baraldi E Daily Telemonitoring of Exhaled Nitric Oxide and Symptoms in the Treatment of Childhood Asthma Am J Respir Crit Care Med 2009 179 93 97 10.1164/rccm.200807-1010OC 18931330

[B14] Asthma UK. Where do We Stand? Asthma in the UK Today 2004

[B15] Alving K Janson C Nordvall L Performance of a new hand-held device for exhaled nitric oxide measurement in adults and children Respir Res 2006 7 67 10.1186/1465-9921-7-67 16626491 PMC1462993

[B16] Khalili B Boggs PB L BS Reliability of a new hand-held device for the measurement of exhaled nitric oxide Allergy 2007 62 1171 4 10.1111/j.1398-9995.2007.01475.x 17845587

[B17] Menzies D Nair A Lipworth BJ Portable exhaled nitric oxide measurement: comparison with the "gold standard" technique Chest 2007 131 410 4 10.1378/chest.06-1335 17296641

[B18] McGill C Malik G Turner SW Validation of a hand-held exhaled nitric oxide analyzer for use in children Pediatr Pulmonol 2006 41 1053 7 10.1002/ppul.20491 16871592

[B19] US Food and Drug Administration http://www.accessdata.fda.gov/cdrh_docs/pdf7/K072816.pdf accessed 17^th ^April 2010

[B20] Aerocrine AB Measuring exhaled NO with NIOX MINO^®^ http://www.aerocrine.com/en/niox-mino/Procedure/(accessed 17^th ^April 2010)

[B21] Gill M Graff GR Adler AJ Dweik RA Validation study of fractional exhaled nitric oxide measurements using a handheld monitoring device J Asthma 2006 43 731 4 10.1080/02770900601031045 17169823

[B22] Tamasi L Bohacs A Bikov A Andorka C Rigo J Losonczy G Horvath I Exhaled Nitric Oxide in Pregnant Healthy and Asthmatic Women Journal of Asthma 2009 46 786 791 19863281 10.1080/02770900903090004

[B23] Miller MR Hankinson J Brusasco V Burgos F Casaburi R Coates A Crapo R Enright P Grinten CP van der Gustafsson P Jensen R Johnson DC MacIntyre N McKay R Navajas D Pedersen OF Pellegrino R Viegi G Wanger J Standardisation of spirometry Eur Resp J 2005 26 319 38 10.1183/09031936.05.00034805 16055882

[B24] Dreborg S Skin tests for the diagnosis of IgE-mediated allergy Allergy 1989 144 supple 10 31 37

[B25] Aerocrine Guide to Interpretation of eNO Values http://www.aerocrine.com/Global/pdf/Interpretation_guide.pdf accessed 17^th ^April 2010

[B26] Gill M Walker S Khan A Green SM Kim L Gray S Krauss B Exhaled nitric oxide levels during acute asthma exacerbation Acad Emerg Med 2005 12 579 86 10.1111/j.1553-2712.2005.tb00910.x 15995087

[B27] Taylor DR Pijnenburg MW Smith AD de Jongste JC Exhaled nitric oxide measurements: clinical application and interpretation Thorax 2006 61 817 827 10.1136/thx.2005.056093 16936238 PMC2117092

[B28] Schneider A Tilemann L Schermer T Gindner L Laux G Szecsenyi J Meyer FJ Diagnosing Asthma in General Practice with Portable Exhaled Nitric Oxide Measurement - Results of a Prospective Diagnostic Study: FENO ≤ 16 ppb better than FENO ≤ 12 ppb to rule out mild and moderate to severe asthma Respiratory Research 2009 10 64 10.1186/1465-9921-10-64 PMC2660901 19254389

